# Millet Could Be both a Weed and Serve as a Virus Reservoir in Crop Fields

**DOI:** 10.3390/plants9080954

**Published:** 2020-07-28

**Authors:** György Pasztor, Zsuzsanna Galbacs N., Tamas Kossuth, Emese Demian, Erzsebet Nadasy, Andras P. Takacs, Eva Varallyay

**Affiliations:** 1 Plant Protection Institute, Georgikon Faculty University of Pannonia, Deák Ferenc Street 17, 8360 Keszthely, Hungary; pasztor018@georgikon.hu (G.P.); nadasyne@georgikon.hu (E.N.); a-takacs@georgikon.hu (A.P.T.); 2 Molecular Plant Pathology Group, Agriculture Biotechnology Research Institute, National Research and Innovation Center, Szent-Gyorgyi A Street 4, 2100 Godollo, Hungary; galbacs.zsuzsanna@abc.naik.hu (Z.G.N.); kossuthtamas96@gmail.com (T.K.); demian.emese@abc.naik.hu (E.D.)

**Keywords:** millet, weed, virus, small RNA HTS

## Abstract

Millet is a dangerous weed in crop fields. A lack of seed dormancy helps it to spread easily and be present in maize, wheat, and other crop fields. Our previous report revealed the possibility that millet can also play a role as a virus reservoir. In that study, we focused on visual symptoms and detected the presence of several viruses in millet using serological methods, which can only detect the presence of the investigated pathogen. In this current work, we used small RNA high-throughput sequencing as an unbiased virus diagnostic method to uncover presenting viruses in randomly sampled millet grown as a volunteer weed in two maize fields, showing stunting, chlorosis, and striped leaves. Our results confirmed the widespread presence of wheat streak mosaic virus at both locations. Moreover, barley yellow striate mosaic virus and barley virus G, neither of which had been previously described in Hungary, were also identified. As these viruses can cause severe diseases in wheat and other cereals, their presence in a weed implies a potential infection risk. Our study indicates that the presence of millet in fields requires special control to prevent the emergence of new viral diseases in crop fields.

## 1. Introduction

Millet is used to feed animals and is a popular alternative cereal. However, as an outcome of recent intensification of crop production, it has also become a dangerous weed, controlled only with difficulty. Herbicides, based on chloraminothiazine, efficiently repress the growth of dicotyledonous weeds in crop fields and leave monocotyledonous millet without a competitor [1,2,3,4]. Currently, only one of its species, *Panicum miliaceum subsp. miliaceum*, is cultivated, but nearly 50 other species are listed as weeds. The results of a recent national weed survey in Hungary revealed that *P. miliaceum* was the seventh-most prevalent weed, representing 1.44% of the weeds present in the investigated fields. Moreover, the presence of other *Panicum* species (*P. capillare, P. ruderale, and P. dichotomiflorum*) was also detected [5]. The germination of maize and millet occurs at the same time, but, with a better transpiration coefficient, lower moisture demand, and an ability to successfully compete for macronutrients, millet overtakes maize in a monoculture and causes substantial damage and yield loss [5,6,7,8,9,10].

Millet can be infected by several viruses. To investigate its role as a potential virus reservoir in our previous work, we surveyed the natural viral infection of millet using DAS ELISA and found that 19 of the 45 samples were virus infected with wheat streak mosaic virus (WSMV), wheat dwarf virus (WDV), barley stripe mosaic virus (BSMV), barley yellow dwarf virus (BYDV), and brome streak mosaic virus (BStMV), either alone or in combination [11].

While serological testing can only reveal the presence of the investigated pathogen, new metagenomics-based studies can reveal the entire virome of the samples and describe the presence of all of the presenting viruses, even if their presence could have not been anticipated [12]. High-throughput sequencing (HTS) offers a technical background for such studies. During viral infection, small interfering RNAs (siRNA) having identical sequences to the infecting viruses are produced by the RNAi-based defense system of the plants [13]. As a result, small RNA (sRNA) HTS has become a widespread virus diagnostic method [14]. Although the host RNAi works efficiently against the invading viruses, it has been shown that siRNAs predominantly arise from the highly structured single-stranded viral RNA [15], and the small RNA-omics approach is not always able to fully and reliably reconstruct the full genome of the infecting virus, especially if it is present in mixed infection [13]. Using the sRNA HTS virome of wheat and barley led to the first description of several viruses [16,17], highlighting the importance of these type of studies.

While crops are widely surveyed for the presence of infecting viruses, knowledge about the viromes of natural flora remains limited. A pioneering geometagenomics study investigating the impact of agriculture on the distribution and prevalence of viruses reached the conclusion that virus prevalence is greater in cultivated areas and that agriculturally important viruses also infect uncultivated plant species [18]. The importance of endemic or invasive weeds in the distribution, prevalence, and diversity of viruses remains, to date, largely uninvestigated.

WSMV, the type member of the *Tritimovirus* genus of the Potyviridae family, is a devastating pathogen in wheat-growing regions globally and was diagnosed in Hungary in the 1980s [19]. Its seed transmission is low, but WSMV can easily spread by a polyphagous wheat curl mite (WCM—*Aceria tosichella*) vector [20]. The host range of the virus and the vector is wide, including millet and grass, even perennial ones, which can overwinter [21,22]. It is hypothesized that WSMV together with its mite vector was introduced to America during germplasm introduction from the Black Sea region in the last century [23].

Barley yellow striate mosaic virus (BYSMV) is a cytorhabdovirus, having a negative single-stranded RNA genome and first being detected in its planthopper vector (*Laodelphax striatellus*) [24]. Although its existence was discovered some time ago, the complete genome of BYSMV was only determined using sRNA HTS and RT-PCR in 2015 [25]. In addition to its original description in Italy and France [24], BYSMVs have been described mainly in Asia—Turkey [26], Iran [27,28], Syria [29], and China [30]—and in Morocco [31]. However, no reports have been made to date about BYSMV in Hungary. The description of BYSMV is based mainly on symptoms and serological tests. With the availability of the sequence information, it was found that BYSMV shares a very high sequence identity with maize sterile stunt virus [32], and thus they should only be considered different strains of the same virus species. BYSMV has also been described in millet [28].

Barley virus G (BVG), a recently described polerovirus, was first found in South Korea in barley [33]. Subsequently, it was reported in Proso [34] and foxtail millet [35], and also in barley and oats in Australia [36]. It has also been reported in the Netherlands, where it was identified in an imported symptomatic switchgrass [37].

In this work, we further investigated the possible virus reservoir role of millet grown as a volunteer weed at two locations using sRNA HTS as an unbiased diagnostic method to be able to detect infection with any viruses. The results confirmed the prevalent presence of WSMV. Moreover, we diagnosed BYSMV and BVG for the first time in Hungary.

## 2. Results and Discussion

### 2.1. sRNA HTS of Millet Populations at Two Locations

We sampled *Panicum miliaceum* plants at two locations in western Hungary near lake Balaton (Figure S1). Millet was grown as a volunteer weed at the side of maize fields and showed typical virus-specific symptoms. The sampled places were the corners of moist maize fields characterized by abundant weeds, where several perennial monocotyledonous plants were growing a short distance outside of the field. At “Budos-arok” (BA), potato had been grown during the previous year, while at “Ujmajor-susnyas” (US), wheat had been grown. Random samples from plants showing different symptoms were collected (Figure S2) and one sequencing library from each location; BA and US were sequenced using the Illumina platform. Virus diagnostics were initially based on the sequenced 19.5 and 31.7 million sRNA reads (Table S2). The presence of any virus-specific contig or coverage of the viral genome greater than 60% was set as a threshold for the presence of a specific virus (Table S3). Based on this analysis, the presence of five plant-infecting viruses was revealed. WSMV, BYSMV, yellow oat-grass mosaic virus (YOgMV), and oat necrotic mottle virus (ONMV) were detected at both sites, and barley virus G (BVG) seemed to be present only at US. In the case of YOgMV and ONMV, in contrast to the presence of several virus-specific contigs and a high number of normalized redundant reads, coverage of the viral genome was very low. YOgMV and ONMV are close relatives of WSMV, being rymoviruses of the Potyviridae family. To test if contigs annotated as these viruses were WSMV contigs originating from a conserved part of the genome, we reannotated YOgMV- and ONMV-specific contigs. The results reveal that they had more than 95% similarity with the WSMV reference genome and could be annotated as WSMV, thus proving that their detection was a false positive and further validating the presence of only WSMV, BYSMV, and BGV.

### 2.2. Millet Weed Is Highly Infected with WSMV

**Figure 1:**
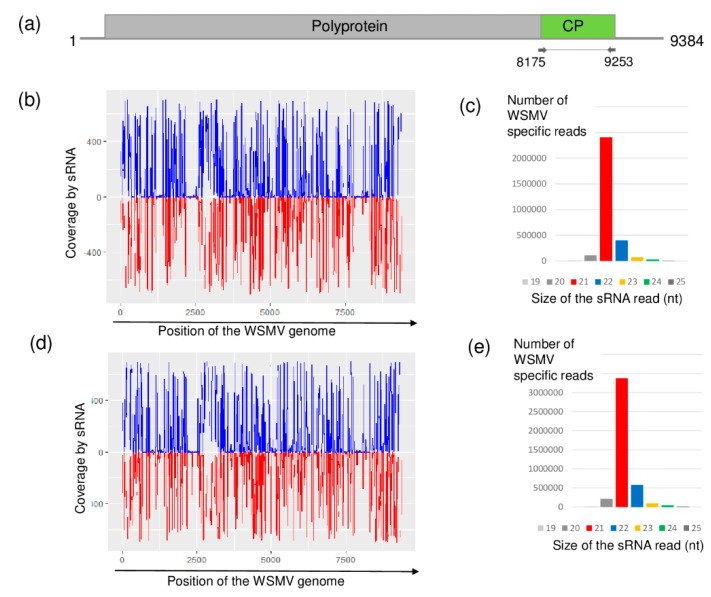
Results of the wheat streak mosaic virus (WSMV) detection by sRNA high-throughput sequencing (HTS). (**a**) Genome organization of WSMV, highlighting its coding regions and the position of the primers that were used in the RT-PCR. (**b**,**d**) show coverage of the genome by the sequenced virus-specific sRNA reads at Budos-arok (BA) and Ujmajor-susnyas (US), respectively. The blue color shows sRNA in sense, while the red color shows sRNAs in antisense orientation. (**c**,**e**) show the size distribution of the sequenced, WSMV-specific sRNA reads at BA and US, respectively.

The presence of WSMV was detected at both locations. WSMV originated, mainly 21 nt long siRNAs of both sense and antisense orientation, were evenly distributed along the viral genome, but the coverage of the genome was not 100% (Figure 1).

Using RT-PCR analysis, amplifying the coat protein (CP) coding region of the WSMV genome revealed that most of the sampled individuals were infected with WSMV. Seven and nine individuals at BA and US were found to be infected, respectively (Figure S3).

**Figure 2:**
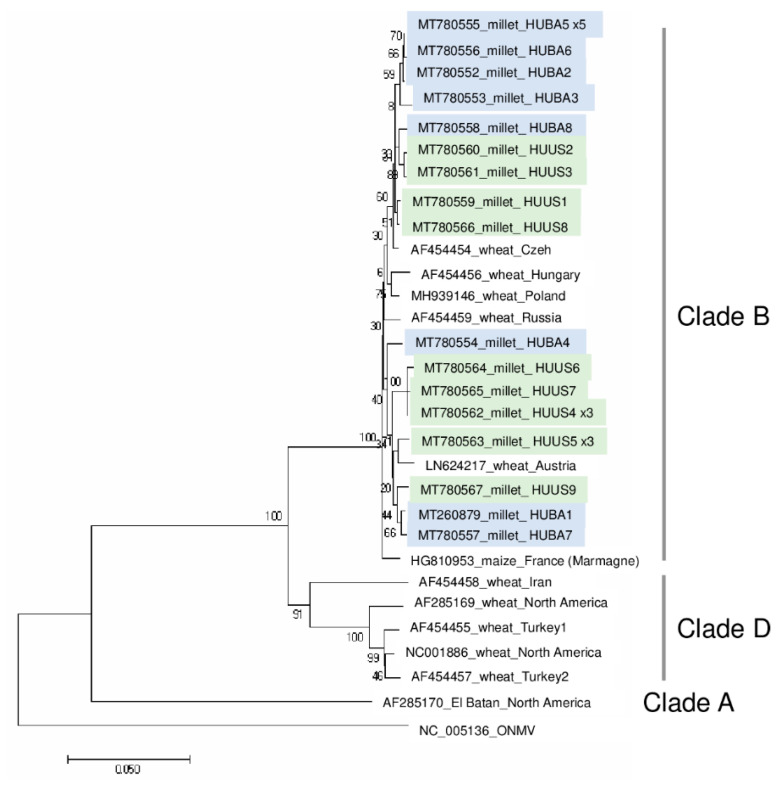
Phylogenetical analysis of the wheat streak mosaic virus strains originated from BA and US. (ONMV—Oat necrotic mottle virus).

According to the sequenced siRNAs, several variants were present, which explains why we cloned and sequenced 13 clones from each location, resulting in eight and nine slightly different variants (named according to their place of origin): WSMV_HUBA1-HUBA8 and WSMV_HUUS1-HUUS9. (HUBA5 was present five times, while HUUS4 and HUUS5 was sequenced three times.) Sequence comparison showed that they all cluster with strains in Clade B (Figure 2).

The most characteristic feature of the WSMV clades is the lack of a single codon within the CP cistron, resulting in a lack of the Gly2761 codon within the CP coding region of the polyprotein in Clade B. This characteristic feature is present in all of the sequenced variants as in the previously sequenced Hungarian strain [23] and in all of the European strains, reported from Czech Republic and Austria [23], Germany [38], Slovakia [39], and Poland [40]. Furthermore, it was shown recently in the Czech Republic that WSMV infects perennial grasses, barley and oat, at field margins, and these strains also belong to Clade B [21,38]. At US, the infection was higher; with the exception of one plant, all of the sampled millet was infected with WSMV. At this location, wheat was grown during the previous year. The source of infection could be WSMV present in the natural flora in overwintering or perennial grasses or it could originate from the wheat cultivar grown there during the previous year. However, as the sequenced strains differed only slightly (they are more than 97% similar to each other) and they are not clusters according to the sample collection places, each origin is possible. Unfortunately, we do not know which wheat variety was cultivated here, and hence judge this hypothesis without further experiments.

Our knowledge about millet as a host of WSMV is scarce, as millet has not been included in detailed host range studies either of crops or perennial grasses [23,41]. However, its high infection rate found in our study raises the possibility of its WSMV reservoir origin. 

### 2.3. Different Strains of BYSMV Were Present at the Two Locations

**Figure 3:**
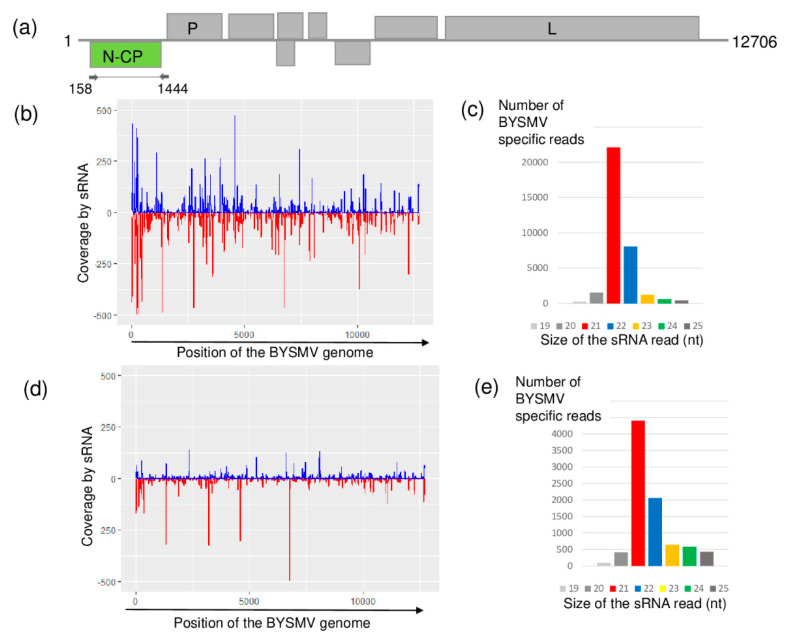
Results of the barley yellow striate mosaic virus (BYSMV) detection by sRNA HTS. (**a**) Genome organization of BYSMV, highlighting its coding regions and the position of the primers that were used in the RT-PCR. (**b**,**d**) show coverage of the genome by the sequenced virus-specific sRNA reads at Budos-arok (BA) and Ujmajor-susnyas (US), respectively. The blue color shows sRNA in sense, while the red color shows sRNAs in antisense orientation. (**c**,**e**) show the size distribution of the sequenced, BYSMV-specific sRNA reads at BA and US, respectively.

Although it has not previously been described in Hungary, according to the sRNA HTS results, both sampled places were infected with BYSMV. BYSMV-derived sRNAs were mainly 21 nt long and equally distributed along the whole genome of this cytorhabdovirus in both libraries (Figure 3). In contrast to this equal distribution, the coverage of the BYSMV genome was just slightly higher than the settled 60% threshold.

**Figure 4:**
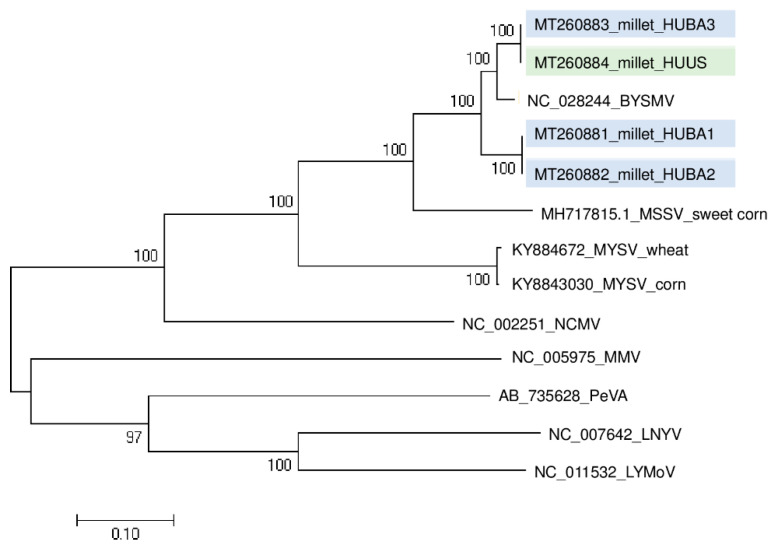
Phylogenetical analysis of the barley yellow striate mosaic virus (BYSMV) strains originated from BA and US. (MSSV—Maize sterile stunt virus, MYSV—Maize yellow striate virus, NCMV—Northern cereal mosaic virus, MMV—Maize mosaic virus, PeVA—Persimmon virus A, LNYV—Lettuce necrotic yellows virus, LYMoV—Lettuce yellow mottle virus).

The RT-PCR validation test of the sampled plants, amplifying the CP coding region of the virus, revealed the presence of BYSMV in three (at BA) and one (at US) plant(s) (Figure S4). (The sequenced variants were named according to their place of origin and numbered.) BYSMV_HUUS and BYSMV_HUBA3 were found to be identical and different from BYSMV_HUBA1 and BYSMV_HUBA2 (Figure 4).

This means that slightly different variants were present, which suggests they can be distributed not only at one location but also at a close distance. Sequence information about the BYSMV CP coding region is only available from the reference genome and not from the variants of different geographical origins. Hence, their phylogenetical analyses were unable to help us hypothesize about the original source of infection.

For more detailed analysis of the phylogenetical origin of the new Hungarian BYSMV strains, deducting sequences from the sRNA HTS would be straightforward. Unfortunately, however, although the sRNA reads are evenly distributed in the genome, the coverage, which was just above 60%, does not allow us to directly undertake this comparison. This feature; low coverage of the viral genome, could be characteristic for the cytorhabdoviruses because, during the comparison of sRNA HTS and RNAseq, Pecman and colleagues found that the presented cytorhabdovirus could be detected only by RNAseq due to the low number of short reads [42]. Sequencing of the RdRp coding region of these strains is needed in the future to allow hypotheses to be made about their origin.

### 2.4. BVG, Which Had Not Previously Been Described in Hungary, Is Present at US 

**Figure 5:**
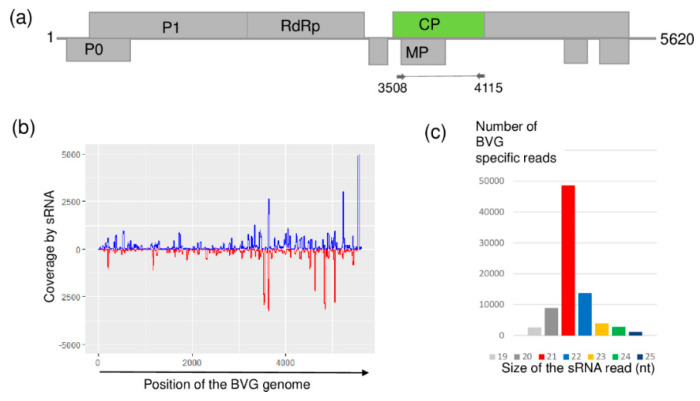
Results of the barley virus G (BVG) detection by sRNA HTS. (**a**) Genome organization of BVG, highlighting its coding regions and the position of the primers which were used in the RT-PCR. (**b**) shows coverage of the genome by the sequenced BVG-specific sRNA reads. The blue color shows sRNA in sense, while the red color shows sRNAs in antisense orientation. (**c**) shows size distribution of the sequenced, BVG-specific sRNA reads.

At US, sRNA HTS showed the presence of BVG, a recently described polerovirus [33]. BGV-originated small RNAs were mainly 21 nt long and covered almost all of the viral genome, reaching 98% coverage (Figure 5).

RT-PCR validation with BVG-specific primers, amplifying the CP coding region, revealed that only one plant (i.e., plant 18) was infected with the virus (Figure S5).

**Figure 6:**
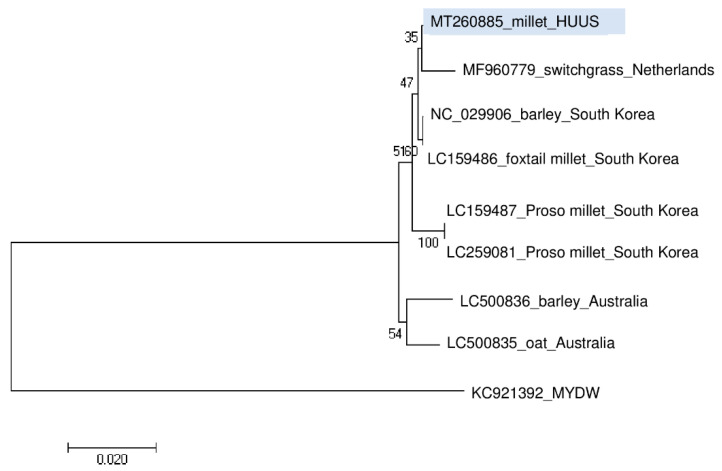
Phylogenetical analysis of the barley virus G (BVG) strain originated from US. (MYDW—Maize yellow dwarf virus).

Phylogenetic analysis of the BVG_HUUS showed that it clusters distantly from the strains reported from Australia and the Proso millet isolates from South Korea. Its closest homologue is the BVG strain from switchgrass (Figure 6).

To the best of our knowledge, with the exception of the report from the Netherlands, BVG has not been reported in Europe. It is therefore surprising that we found this virus in millet weed; it is difficult to explain its presence or speculate about its origin. Plant 18, which was infected with the virus, did not show any specific symptom (Figure S2). Although the symptoms of BVG remain unclear, it is possible that it may affect cereal yields and biomass production [37].

## 3. Materials and Methods 

### 3.1. Plant Material and Sample Preparation

Samples were collected from two locations in Hungary in August 2019 (Figure S1). At both BA and US, 10 individual *Panicum miliaceum* plants were sampled randomly, representing different symptoms. We collected leaves together with root and millet seeds if it was possible (see Table S1 for detailed information, and Figure S2 for photos of the sampled individuals). From the samples, we extracted RNA using a phenol-chloroform method [43]. Briefly, frozen plant material was homogenized in an ice-cold mortar, suspended in 650 µL of extraction buffer (100 mM glycine, pH 9.0, 100 mM NaCl, 10 mM EDTA, 2% SDS, and 1% sodium lauroylsarcosine), mixed with an equal volume of phenol, and centrifuged for 5 min. The aqueous phase was treated with equal volumes of phenol, chloroform, and isoamyl-alcohol (25:24:1), centrifuged for 5 min and, after subsequent treatment of the upper phase with chloroform:isoamyl-alcohol (24:1) and centrifugation for 5 min, the upper phase was precipitated with ethanol and then re-suspended in sterile water. Obtained total nucleic acid extracts were stored at −70 °C until used.

### 3.2. sRNA Library Preparation and Sequencing

RNA pools of the individuals were prepared by mixing equal amounts of RNA from different organs of the same plant. Pools according to the location were prepared with the same strategy by mixing equal amounts of RNA originating from different individuals representing all of the sampled plants at the particular location. This pooling strategy allowed for the detection of any virus present in any of the sampled individual plants. These pools were used for sRNA library preparation (2 libraries in total). We purified sRNA fractions with the size separation on polyacrylamide gel-electrophoresis and followed our in-house-modified protocol [44] based on TruSeq Small RNA Library Preparation Kit (Illumina, San Diego, CA, USA). Two sRNA libraries were sequenced using a single index on a HiScanSQ by UD-Genomed (Debrecen, Hungary) (50 bp, single-end sequencing). FASTQ files of the sequenced libraries were deposited in the GEO and can be accessed through series accession number GSE147185.

### 3.3. Pipeline for Data Evaluation of HTS Results (Bioinformatics)

For virus diagnosis, the bioinformatics analysis of the HTS results was carried out using a CLC Genomic Workbench (Qiagen, Hilden, Germany). After the trimming and quality control of the reads, a QC report was prepared by applying embedded protocols in the CLC Genomic Workbench. Longer contigs were built from the non-redundant reads using an assembler of CLC (de novo assembly using default options: word size 20, bubble size 50, and simple contig sequences and min 35 nt lenght) (see Table S2 for initial statistics of the reads). The contigs were annotated using the blastn algorithm with default options (thread 1, word size 11, match 2, mismatch 3, gap cost existence 5, extension 2) and the NCBI Plant hosted Viral Reference genomes (downloaded at 0307 2019). For the viruses that were represented by at least one contig, the reads were directly mapped to the reference genome and counted with and without redundancy (using the map to the reference command allowing 1 mismatch). The number of normalized redundant reads (read/1 million reads—RPM) was calculated from the mapped redundant reads and the number of total sequenced reads. Based on the mapping, a consensus sequence was prepared and used to calculate the coverage (%) of the viral genome (Table S3). If at least two parameters of any of (i) the presence of any virus specific contigs, (ii) the number of normalized redundant virus specific read >200, or (iii) the coverage of the virus genome >60% were fulfilled, we further investigated the presence of the virus first by reannotating the viral specific contigs and then by RT-PCR, an independent virus diagnostic method.

### 3.4. Virus Diagnostics by RT-PCR

Pooled RNA extracts representing each sampled plant, and the plantation pool that was used for the sRNA HTS, were used as templates for cDNA synthesis by RevertAid First Strand cDNA Synthesis Kit (Thermo Fisher Scientific, Waltham, MA, USA) with random primers according to the manufacturer’s instructions. Viruses were diagnosed by RT-PCR performed with Q5 Hot Start High-Fidelity DNA Polymerase (New England Biolabs, Ipswich, MA, UK) (the primers used to amplify viral parts together with the used annealing temperatures and cycling parameters are provided in Table S4). For Sanger sequencing. PCR products were purified using GeneJET Gel Extraction Kit (Thermo Fisher Scientific), cloned into GeneJET (Thermo Fisher Scientific) and sequenced. Sequences were deposited into GenBank (for GenBank accession numbers, see Table S5 and Figures S3–S5).

### 3.5. Phylogenetic Analysis of the Detected Viral Strains

For phylogenetic analysis of viral sequences, we used MEGA7 [45]. Evolutionary history was inferred using the neighbor-joining method [46]. The percentage of replicate trees in which the associated taxa clustered together in the bootstrap test (500 replicates) is shown next to the branches of the phylogenetic trees [47]. The evolutionary distances were computed using the Jukes–Cantor method [48] and are presented as the number of base substitutions per site.

## 4. Conclusions

Our pilot study shows that sRNA HTS can reveal new insights of investigated plants and lead to the description of viruses, BYSMV and BGV in our case, even if they have not been previously described or are not anticipated at the surveyed location. In addition, we found a high infection rate with WSMV supporting the hypothesis of Schubert and colleagues that viruses can establish themselves in seed production areas and survive on perennial grasses growing at field boundaries [38]. This highlights the need for, and importance of, detailed virus diagnostic surveys to help describe the existence, and also the possible variants, of the presenting viruses, which should be taken into account during resistance breeding to avoid the damage caused by resistance-breaking new variants.

Our results suggest that, in addition to its direct competitive effect, millet could serve as a virus reservoir and might play a role in the spread of some cereal viruses. Thus, the control of millet weed, as well as the prevention of its overwintering and volunteer growth, is of paramount importance. However, to determine the importance and frequency of this type of virus distribution, further studies are needed. Insect vectors play a pivotal role in virus spread. Analysis of their virome, which is a new area of investigation [49], can add new insights to this field. As weeds can both host aphid vectors and viruses, they could play a key role in virus distribution [50]. To determine the spread of this mechanism, and identify the agronomically important viruses that could use this strategy further, directed surveys should be conducted in the future. 

## Supplementary Materials

The following are available online at https://www.mdpi.com/2223-7747/9/8/954/s1, Figure S1: Map of the sample collection sites. Figure S2: Photos of the sampled millet individuals collected at (**a**) BA and (**b**) US. Figure: S3: Validation of the presence of WSMV by RT-PCR. Figure S4: Validation of the presence of BYSMV by RT-PCR. Figure S5: Validation of the presence of BVG by RT-PCR.; mdpi.com/xxx/s2 Table S1: Summary of sampling of millets at two different locations. Table S2: Initial statistics of the small RNA HTS. Table S3: Bioinformatics analysis of the sequenced libraries. Table S4: Sequences of the primers used for virus detection in RT-PCR together with the PCR cycling parameter. Table S53: GenBank identifiers of the amplified and sequenced virus specific products.
