# Supplementary material for: Millet Could Be both a Weed and Serve as a Virus Reservoir in Crop Fields
Source: Plants (Basel). 2020 Jul 28;9(8):954. doi: 10.3390/plants9080954 (PMC7463774; doi:10.3390/plants9080954)
Supplement: Supplementary file 1 [file plants-09-00954-s001.zip › Pasztor_Galbacs et al_Plants_2020_S1_ve.docx]

Supporting S1 to

Millet could be both a weed and serve as a virus reservoir in crop fields

György, Pasztor^x^, Zsuzsanna, N. Galbacs^x^, Tamas, Kossuth, Emese, Demian, Erzsebet, Nadasy, Andras P., Takacs, Eva, Varallyay*

*****Correspondence: [varallyay.eva@abc.naik.hu](mailto:varallyay.eva@abc.naik.hu)


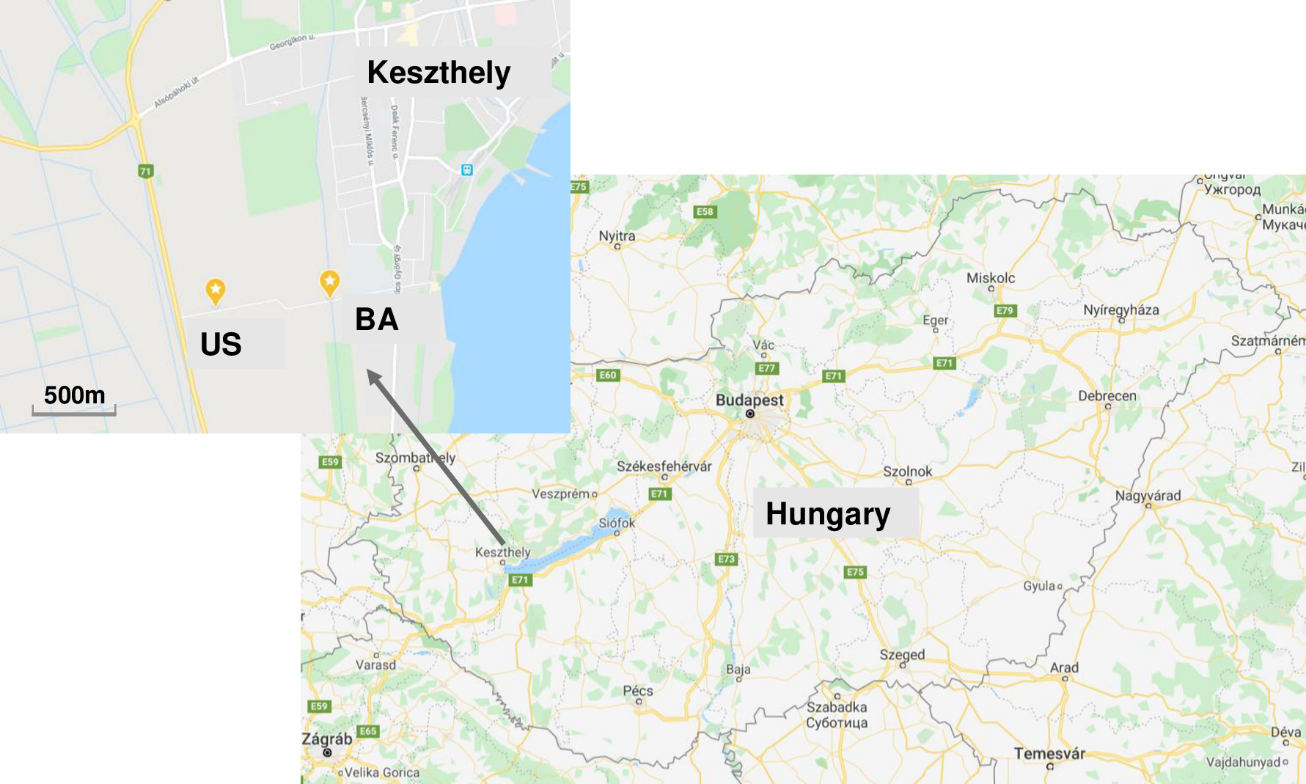


**Figure S1.** **Map of the sample collection sites.**


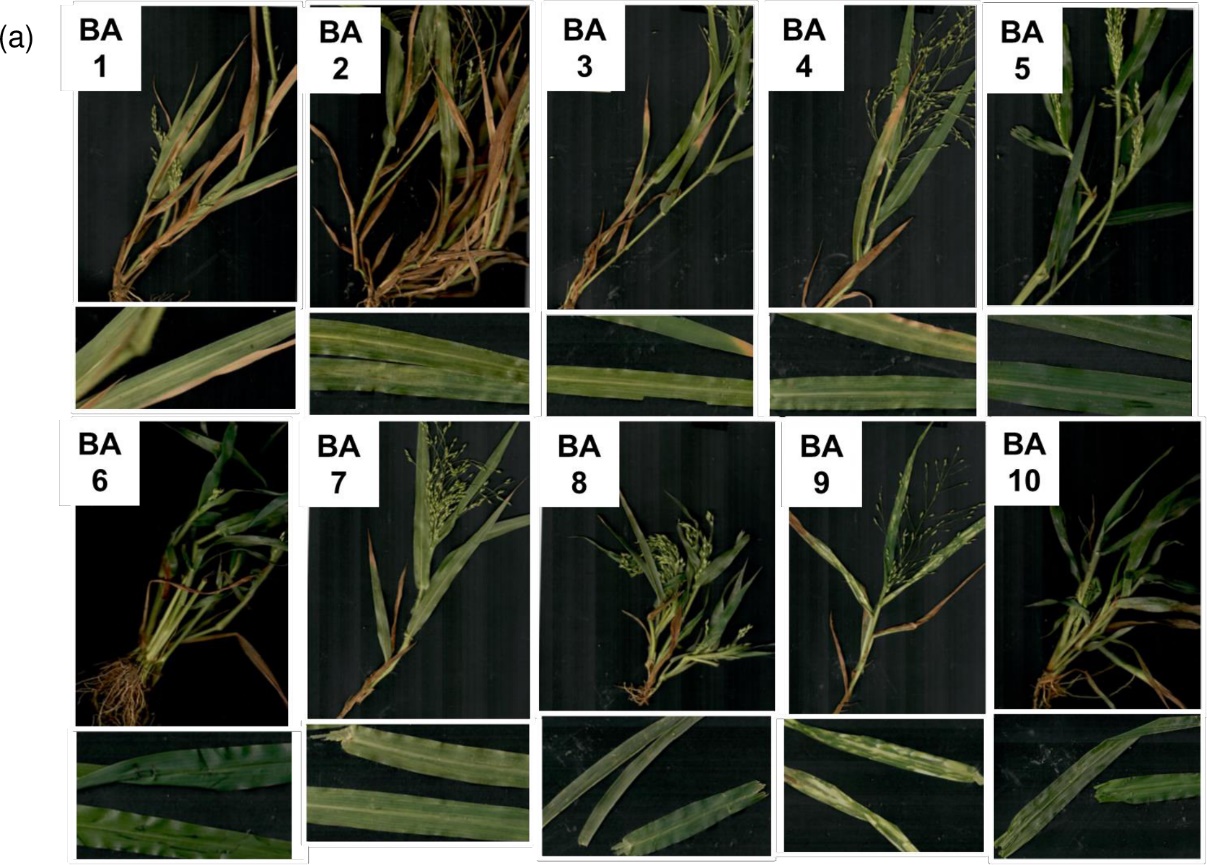


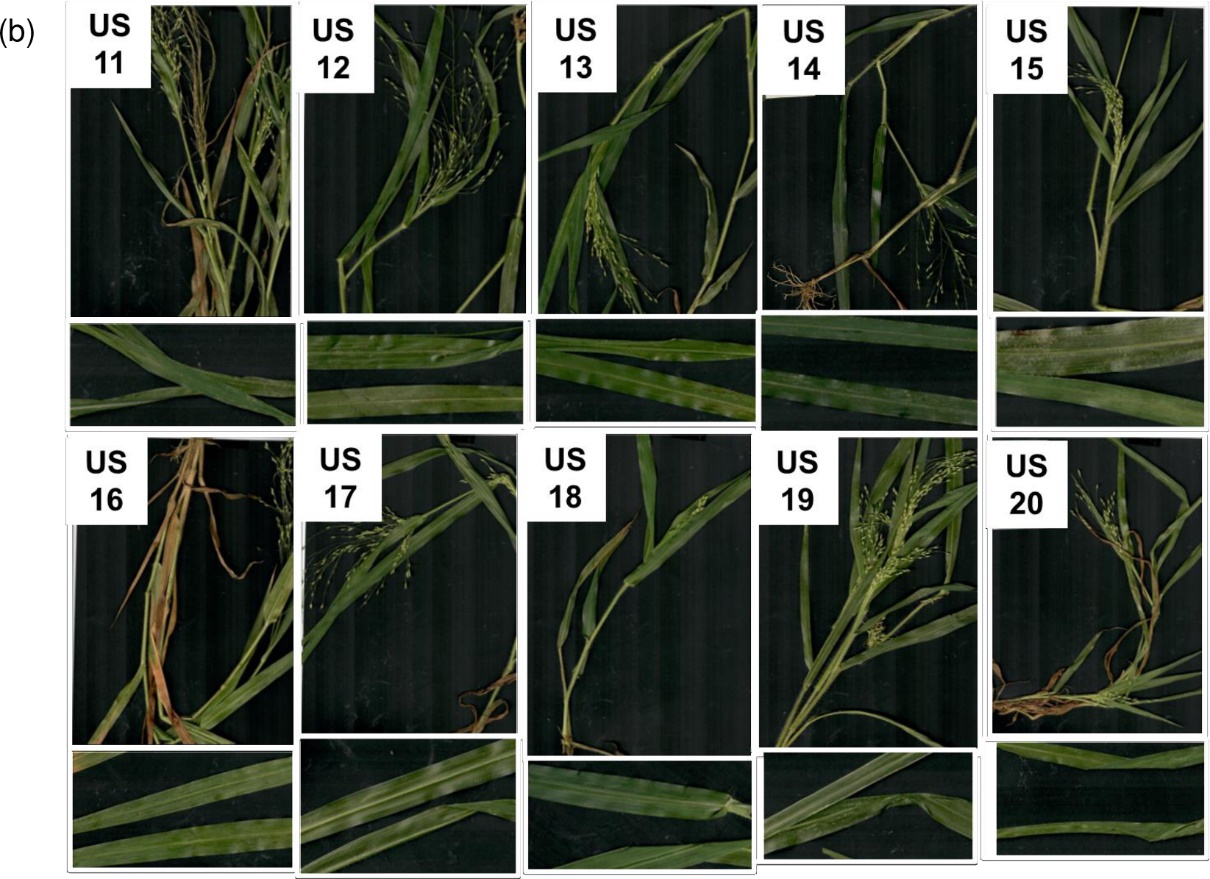


**Figure S2. Photos of the sampled millet individuals collected at (a) BA and (b) US.**


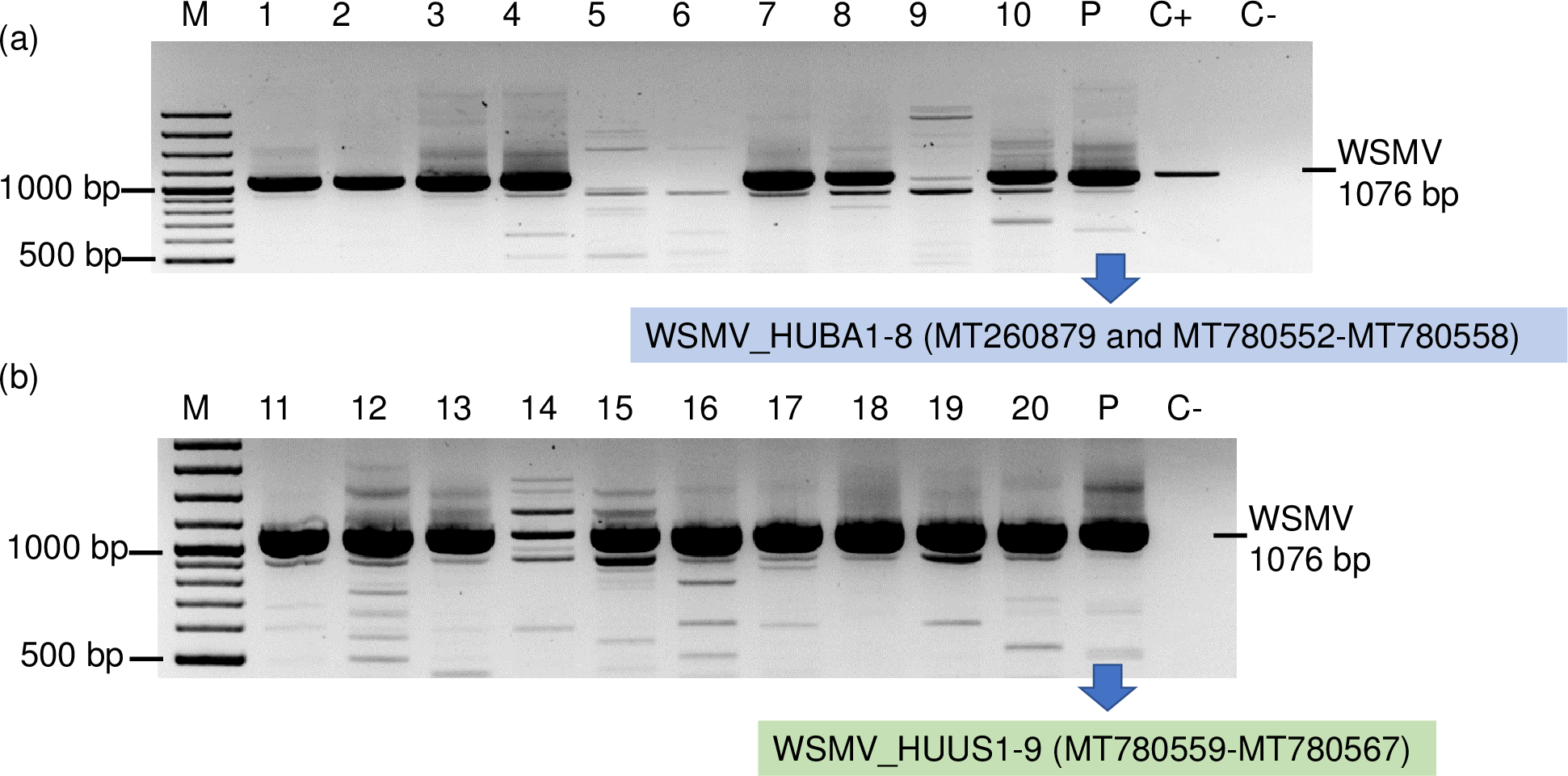


**Figure S3**. **Validation of the presence of WSMV** **by RT-PCR** in the sampled individuals (a) at BA and (b) at US. P marks a reaction where the RNAs of the individuals were tested as a pool. Coloured boxes mark the origin of the products which sequence was deposited into the GenBank after amplification with a proofreading DNA polymerase (Q5 from New England Biolabs) and cloning. with their GenBank identification numbers. M: GeneRuler 100 bp Plus DNA ladder (Thermo Fisher Scientific, USA). Sequence of the product in sample #14 showed that it originates from the host.


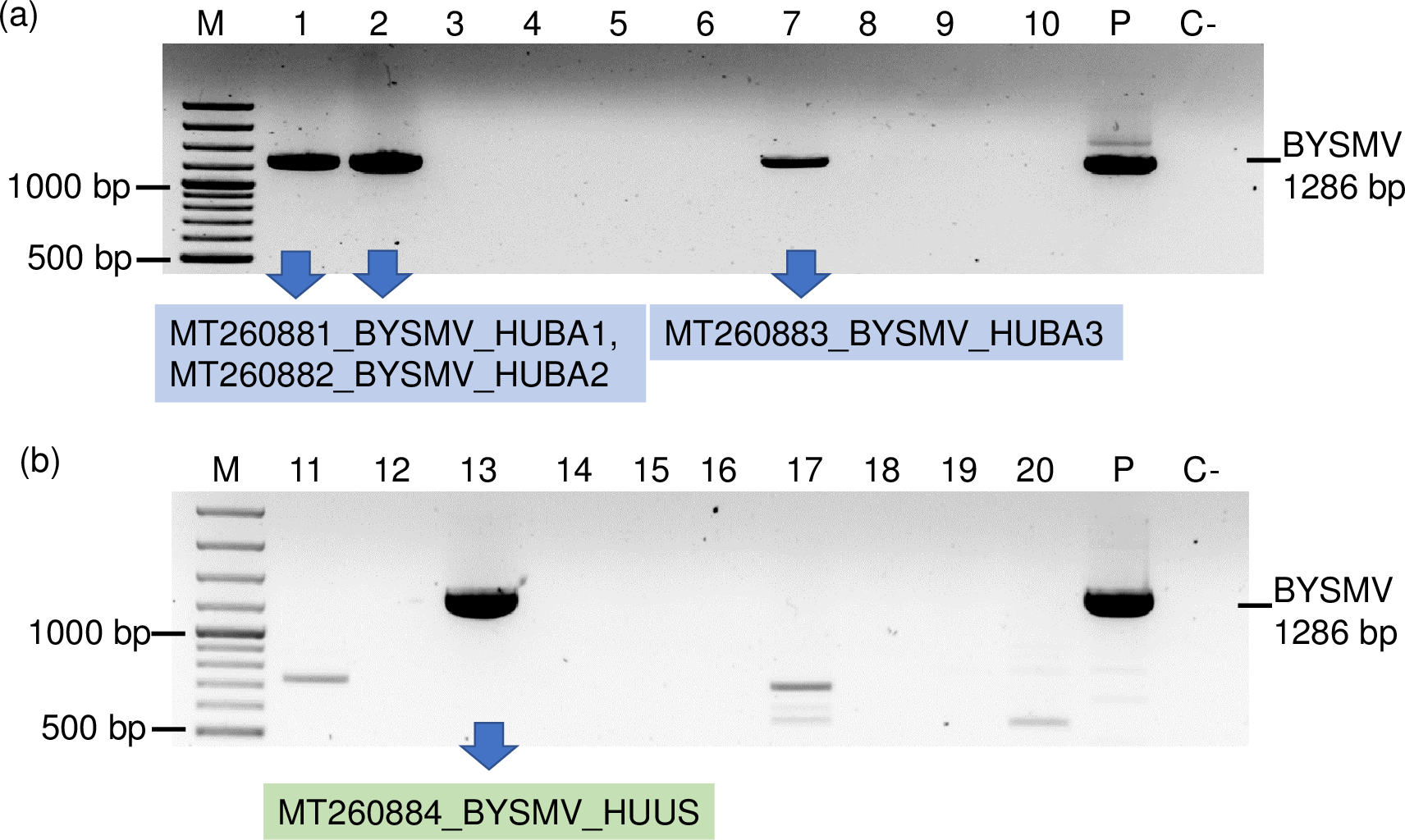


**Figure S4**. **Validation of the presence of BYSMV by RT-PCR** in the sampled individuals (a) at BA and (b) at US. P marks a reaction where the RNAs of the individuals were tested as a pool. Coloured boxes mark the origin of the products which sequence was deposited into the GenBank after amplification with a proofreading DNA polymerase (Q5 from New England Biolabs) and cloning with their GenBank identification numbers. M: GeneRuler 100 bp Plus DNA ladder (Thermo Fisher Scientific, USA).


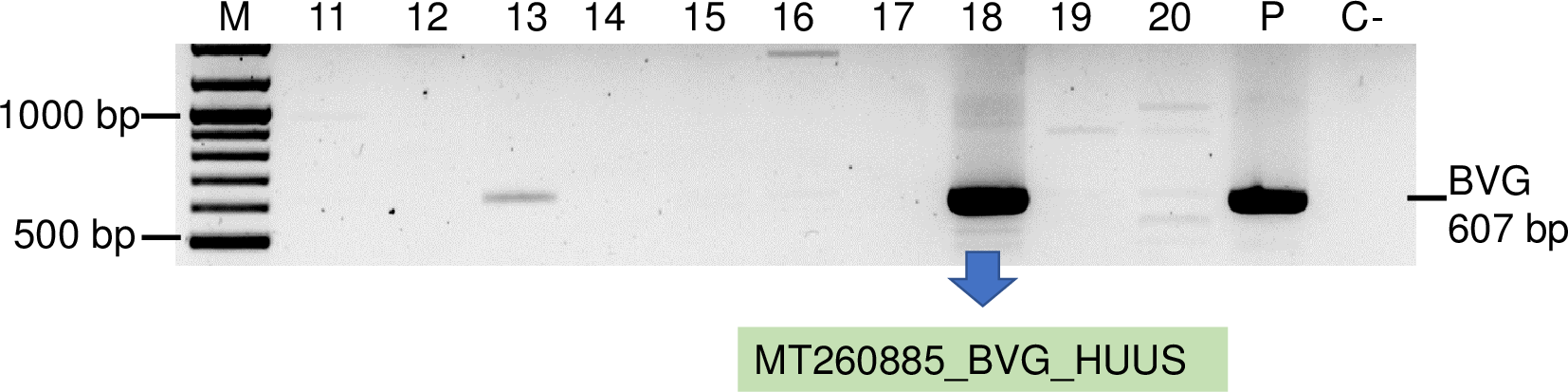


**Figure S5**. **Validation of the presence of BVG by RT-PCR** in the sampled individuals (a) at BA and (b) at US. P marks a reaction where the RNAs of the individuals were tested as a pool. Coloured box marks the origin of the product which sequence was deposited into the GenBank after amplification with a proofreading DNA polymerase (Q5 from New England Biolabs) and cloning with its GenBank identification number. M: GeneRuler 100 bp Plus DNA ladder (Thermo Fisher Scientific, USA). Sequence of the product in sample #13 showed that it originates from the host.
